# SWI/SNF subunit BAF155 N-terminus structure informs the impact of cancer-associated mutations and reveals a potential drug binding site

**DOI:** 10.1038/s42003-021-02050-z

**Published:** 2021-05-05

**Authors:** Mark D. Allen, Stefan M. V. Freund, Mark Bycroft, Giovanna Zinzalla

**Affiliations:** 1 UKRI MRC Laboratory of Molecular Biology, Cambridge, UK; 2 Microbiology, Tumor and Cell Biology (MTC) Department, Karolinska Institutet, Stockholm, Sweden; 3 Present Address: Department of Pharmacology, University of Cambridge, Cambridge, UK

**Keywords:** X-ray crystallography, Cancer therapy

## Abstract

SWI/SNF (BAF) chromatin remodelling complexes are key regulators of gene expression programs, and attractive drug targets for cancer therapies. Here we show that the N-terminus of the BAF155/SMARCC1 subunit contains a putative DNA-binding MarR-like domain, a chromodomain and a BRCT domain that are interconnected to each other to form a distinct module. In this structure the chromodomain makes interdomain interactions and has lost its canonical function to bind to methylated lysines. The structure provides new insights into the missense mutations that target this module in cancer. This study also reveals two adjacent, highly-conserved pockets in a cleft between the domains that form a potential binding site, which can be targeted with small molecules, offering a new strategy to target SWI/SNF complexes.

## Abstract (web summary)

Allen et al. determine crystal structure of the N-terminus of SWI/SNF chromatin remodelling factor subunit BAF155. They identify an interconnected structure of MarR-like, BRCT and chromodomains, visualise the potential effect of cancer-associated missense mutations and suggest a binding site and target for small molecule inhibitors.

## Introduction

mSWI/SNF or BAF complexes are ATP-dependent chromatin regulators^1^. They are combinatorially assembled from 16 subunits encoded by 31 genes into a range of complexes with specific and distinct roles in many key biological processes. Several of the subunits are frequently mutated in a range of cancers. mSWI/SNF complexes have a role in both tumour suppression and oncogenesis, and have emerged as promising targets for cancer therapy^1,2^. The development of small molecules targeting these complexes remains challenging as many of the subunits lack amenable binding sites. The BAF155/SMARCC1 subunit is a component of all complexes identified to date, incorporated either as a homodimer or as a heterodimer with its paralog BAF170/SMARCC2. The BAF155 subunit predominates in stem cells, while the BAF170 subunit is incorporated during differentiation. BAF155 is the only subunit present in the esBAF and ncBAF complexes^3^. BAF155 is a potential therapeutic target for colorectal^4^ and prostate^5^ cancers. Both BAF155 and BAF170 are mutated in several cancers with the worst prognosis, for which no therapies are available. It has been found that cells with mutations in either of the subunits are sensitive to the loss of the other paralog, providing a means of selectively targeting cancers in which these subunits are mutated^6^. This type of synthetic lethal interaction has been exploited to target cancers in which the SMARCA4 subunit is mutated by employing PROTAC degraders directed at its paralog SMARCA2^7^.

**Fig. 1:**
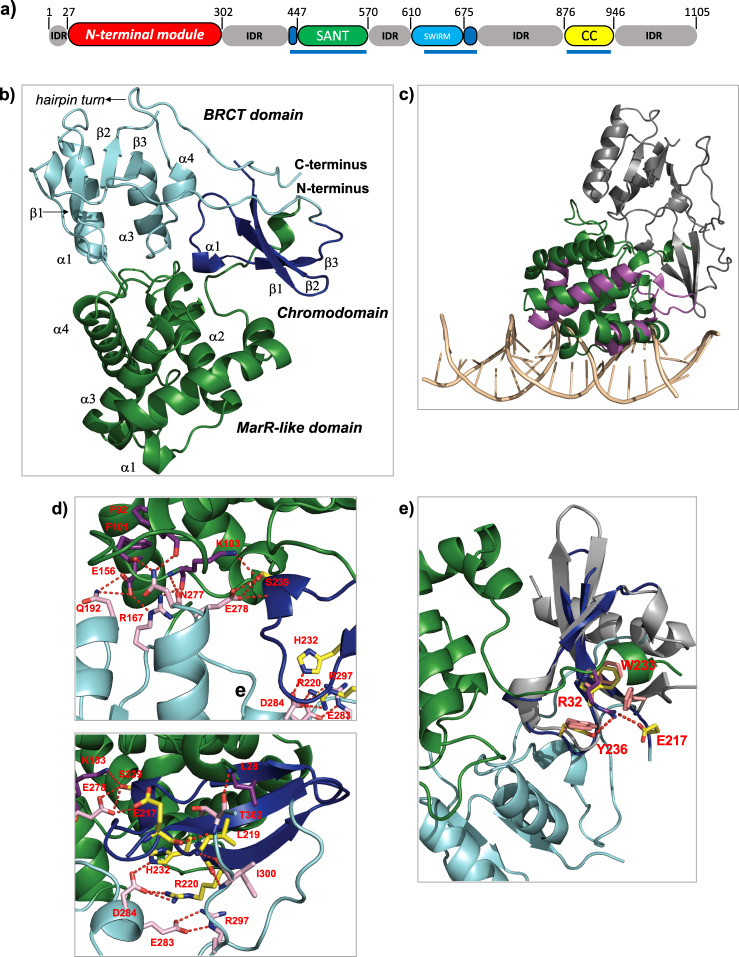
X-ray structure of the N-terminal module of BAF155. **a** Domain organization of BAF155. Blue bars indicate regions visible in the cryo-EM structure (6LTH). **b** Cartoon representation of the structure with BRCT domain in cyan, chromodomain in blue, the MarR-like domain in green. **c** Overlay of the carton representations of the MarR-like domain structure (in green) of BAF155 and of the protein TipAL (in purple, bound to DNA in orange, PBD ID = 2VZ4). **d** Zoom in of the hydrogen-bonding network. Residues in the hydrogen-bonding network are shown in stick representation for the three domains and the C- and the N-termini (pink = BRCT domain, yellow = chromodomain, purple = MarR-like domain). **e** Overlay of the cartoon representation of the chromodomains of BAF155 (in blue) and CBX7 (PDB ID = 4X3K) (in grey). Residues of the aromatic cage are shown in yellow in BAF155 and in pink in CBX7, illustrating the blocking of the methyl-lysine-binding site on BAF155 by Arg32 (in purple).

Only 27% of the residues of BAF155 are visible in the recent cryo-EM structure of the BAF complex^8,9^ (Fig. 1a). These amino acids make extensive contacts with other subunits and offer no targetable binding sites. The rest of the protein is predicted to be intrinsically disordered with the exception of an approximately 250-amino-acid region at the N-terminus (Fig. 1a), which, based on sequence analysis, is expected to contain a chromodomain and a BRCT domain, uniquely having the former in the middle of the latter. No cross-linking between this region and any other BAF subunit has been detected in several proteomics studies. This is similar to what has been observed for the bromodomain of the SMARCA4 subunit and the PHD finger domains of the Dpf2 subunit, both of which mediate interactions with chromatin^9^. Although BAF155 homologues are ubiquitous in eukaryotes, this region is only found in metazoa. The presence of the N-terminal module in these species suggests that its acquisition is linked to more-complex regulation of gene expression in multicellular animals, however, nothing is known about its function. Chromodomains can recognize methylated lysine residues in histones, and BRCT domains are found in many proteins that participate in the DNA damage response, suggesting that this region could play an important role in regulating the activity of the complex. Both of these domains are also being explored as potential small-molecule targets for drug discovery.

We therefore set out to determine the structure of the N-terminus of BAF155 using X-ray crystallography. This shows that the this region contains a distinct structural module where the chromodomain, the BRCT domain and a MarR-like domain are interconnected by a complex hydrogen-bond network. This also reveals the presence of a binding pocket at the interface between the three domains that can be targeted by a small-molecule drug. The structure of the N-terminus allows the assessment of the impact of mutations in cancer that target BAF155 and its paralogue BAF170. This study offers a potential approach to develop cancer therapies.

## Results and discussion

### Overall structure and architectural organization of BAF155 N-terminus

Residues 27–383 of BAF155 were expressed and purified, and their structure determined using X-ray crystallography (Fig. 1b, Supplementary Fig. 1a and Table 1). Residues 4–160 form a four-helix-bundle that resembles the MarR-like helix-turn-helix domain^10^ based on structure homology analysis using VAST:^11^ 43 aligned residues, RMSD for CA atoms of 1.9, score 5.1, *P*-value = 0.0005 for the highest scoring protein (Supplementary Fig. 2a). Interestingly, a homologous domain is also present in the INI1/SNF5/SMARCB1 subunit^12^. MarR domains bind to the major groove of DNA via the second helix of the motif with a β-strand wing also contacting the minor groove (Fig. 1c). The wing is absent in our structure, but residues in α2 are highly conserved as are residues in the adjacent loop between α3 and α4. Both α1 and α4 are longer than in MarR domains and the orientation of α4 differs to allow for interactions with other domains in the module (Fig. 1b). This would block the type of interaction that MarR domains normally make with DNA, although the BAF155 module could interact with distorted DNA structures. Residues 169–287 consist of a BRCT domain with a chromodomain (residues 219–246) inserted between the second and third strands of the canonical fold (Supplementary Fig. 1a). Helix 2 that forms part of a phosphate-binding site in many BRCT domains^13^ is missing (Supplementary Fig. 2). The chromodomain consists of the curved antiparallel three-stranded β-sheet characteristic of this domain. The C-terminal helix that packs across the β-sheet^14^ in many chromodomains is not present in the BAF155 structure; instead hydrophobic residues (Val249, Ile253) in the loop, which links the chromodomain to β2 of the BRCT domain pack onto the sheet (Fig. 1d).

**Table 1:** Data collection, phasing and refinement statistics for SAD (SeMet) structure determination.

	6YXP (Native)^a^	6YXO (SeMet)^a^
Data collection		
Space group	P2_1_	P2_1_
Cell dimensions		
* a*, *b*, *c* (Å)	43.900, 138.322, 56.510	43.810, 136.815, 56.042
α, β, γ (°)	90.0, 110.311, 90.0	90.0, 110.516, 90.0
Wavelength (Å)	0.939273	0.975456
Resolution (Å)^b^	49.49–1.60 (1.70–1.60)	49.01–2.00 (2.10–2.00)
* R*_sym_ or *R*_merge_^b^	0.053 (0.611)	0.069 (0.432)
* I*/σ*I* ^b,c^	99.1 (98.2)	10.9 (2.9)
CC(1/2)^b,d^	0.999 (0.850)	0.997 (0.899)
Completeness (%)^b^		99.5 (99.3)
Anomalous Completeness (%)^b^		97.0 (98.4)
Redundancy^b,e^	6.8 (6.9)	5.4 (5.6)
Anomalous redundancy^b,e^		2.8 (2.8)
Refinement		
Resolution (Å)	1.60	2.00
No. of reflections	82,268	41,388
* R*_work_/*R*_free_^f^	0.1727/ 0.1978	0.1973/ 0.2382
No. of atoms		
Protein	4509	4482
Water	515	337
* B*-factors^g^		
Protein	35.81	43.69
Water	42.70	44.62
R.M.S. deviations^h^		
Bond lengths (Å)	0.006	0.006
Bond angles (°)	0.800	0.628

^a^One crystal for each structure. ^b^Values in parentheses are for highest resolution shell. ^c^Signal to noise ratio of intensities. ^d^CC_1/2_ is the correlation coefficient of the mean intensities between two random half-datasets. ^e^Multiplicity for unique reflections. ^f^5% of reflections were randomly selected for determination of the free R factor, prior to any refinement. ^g^Temperature factors averaged for all atoms. ^h^R.M.S. deviations from ideal geometry for bond lengths and restraint angles.

The individual domains are linked via a network of hydrogen bonds to form a single structural unit in which a highly conserved stretch of amino acids in the α3-α4 loop and α4 of the BRCT domain plays a central role (Fig. 1d). The sidechain of Asn277 hydrogen bonds to residues in the α2-α3 loop of the MarR-like domain. These domains are also linked by hydrogen bonding between Glu156 in α4 of the MarR-like domain and Glu192 at the C-terminus of α1 of the BRCT domain. The sidechain of Glu278 hydrogen bonds to the sidechain and backbone of Ser239 in the small helix between β2 and β3 of the chromodomain. The sidechain of Ser239 also hydrogen bonds to the sidechain of Lys103 linking the chromodomain to the MarR-like domain. Asp284 hydrogen bonds to the sidechains of Arg220 at the start of β1 and His232 at the end of β2 of the chromodomain and the amide of Phe235 in the β2-α1 loop. The BRCT domain is followed by a hairpin turn allowing Arg297 to form a salt bridge with Glu283. The backbone amides of residues Thr300 and Ile302, which are located at the C-terminus of the module, hydrogen bond with the backbone carbonyls of Glu217 and Leu219 at the start of β1 of the chromodomain. Analysis of the sequence of human chromodomains shows that the BAF155 domain clusters with so-called “chromobarrel domains”^14^. This sub family of chromodomains have an additional N-terminal strand that precedes the chromodomain fold: the BAF155 chromodomain does have a chromobarrel fold, but in this case the residues located at the C-terminus of the module form the additional strand. Residues 28–33 pack onto the chromodomain, and a main-chain to main-chain hydrogen bond is formed between Leu28 and Ile300 linking the N- and C-termini of the module.

### A non-functional chromodomain

The N-terminal residues interact with a region of the chromodomain that in others forms an aromatic cage that binds to methylated lysines. Only two of the three residues of the cage, in for example CBX7, are present (Trp233, Tyr236) in the BAF155 chromodomain (Supplementary Fig. 3a), and Arg32 is positioned where the methylated lysine sidechain would interact^14^ (Fig. 1e). As is the case for other chromobarrel domains, the presence of the additional strand prevents the domain from making the types of interaction that polycomb or HP1 family chromodomains make with the residues adjacent to methylated lysines in histone H3. Chromobarrel domains that interact with methylated lysine, such as the MSL3 domain, only contact the modification, without making significant contact with the adjacent amino-acid residues. Therefore, for these types of domain, free methylated lysine has been used^15^ to probe their ability to interact with histone modifications. No binding to free methylysine derivatives (mono-, di- or tri-methylated) to the BAF155 chromodomain was detected using NMR spectroscopy, which has been used to characterize even very weak binding to aromatic cages^15^ (Supplementary Fig. 3b, c). This is not unprecedented as many other chromobarrel domains also do not interact with methylated histones. The domain from TIP60, for example, which is closely related in terms of sequence to the BAF155 chromodomain, lacks the same residue in the aromatic cage, and also has an Arginine residue inserted where the methylated lysine would normally bind^16^ (Supplementary Fig. 3d).

### Identification of a binding pocket at the interface between the three domains

**Fig. 2:**
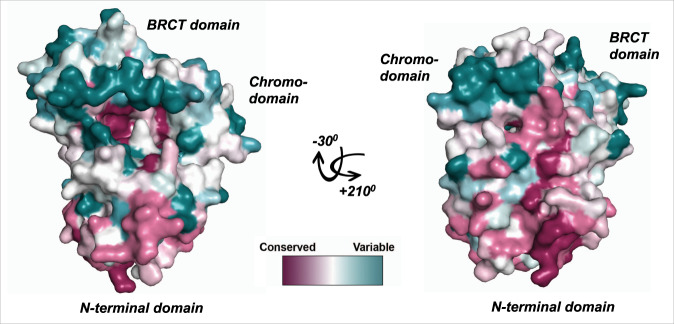
Conservation analysis. Surface representation of the X-ray structure of the N-terminal module of BAF155 coloured by sequence conservation using the program ConSurf.

The architecture of the fold produces a cleft containing two deep pockets at the interface between the domains. Analysis of the sequence conservation using ConSurf^17^ (Fig. 2 and Supplementary Fig. 1a) reveals two highly conserved regions on the surface of the module: the first on the MarR-like domain (Supplementary Fig. 1b) and the second within this cleft. In contrast, the two regions corresponding to the canonical binding sites of chromodomains and BRCT domains are not conserved (Fig. 2 and Supplementary Fig. 1a). Some of the conserved residues in the cleft are part of the network of residues that link the domains, while others such as Asp111 and Asp241 have no structural role, suggesting that this region is functionally important.

Computational analysis using PeptiMap^18^ predicts that the pockets within this cleft could bind to peptides (Supplementary Fig. 4), suggesting that this region mediates protein–protein interactions (PPIs). As the cleft is adjacent to the conserved region on the MarR-like domain, it is possible that N-terminal module of BAF155 participates in an interaction involving both protein and nucleic acid components.

### Analysis of the impact of cancer-associated mutations

**Fig. 3:**
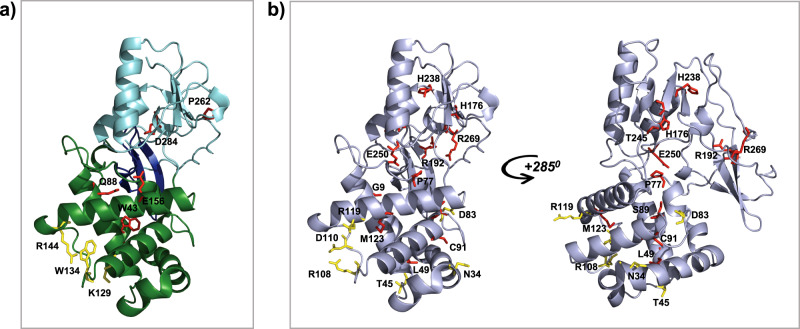
Deleterious mutations. **a** Cartoon representation of the X-ray crystal structure of the N-terminal module of BF155 with residues labelled in red for the structurally damaging mutations, and in yellow for mutations predicted to affect functionally important residues. **b** Mutations in BAF170 shown on a cartoon representation of a homology model with residues highlighted as in panel (**a**).

With the structure in hand we examined sequence variants within the module that have been identified in cancer-genome sequencing studies to try and distinguish between driver and passenger mutations. Six missense mutations of BAF155 identified in patient samples that map to the module are predicted to destabilise its structure as judged by Missense3D^19^ (Supplementary Table 1). Three of these are of residues (Asp284 and Glu156) that form salt bridges in the hydrogen-bonding network linking the domains together. Three mutations are of highly conserved surface residues (K129T, W134L and R144Q) located in the MarR-like domain (Fig. 3a and Supplementary Fig. 1b), which are likely to affect the function of the N-terminal module (Fig. 3a and Supplementary Fig. 1b). As the N-terminal module is also present in the BAF170 paralogue with 66% identity, we used our structure to generate a homology model and carried out the same analysis (Supplementary Table 2). In BAF170, 14 mutations are predicted to be damaging to the fold. As seen for BAF155, several of these are of residues in the hydrogen-bond network linking the domains together (Fig. 3b). Eleven mutations affecting highly conserved surface residues were identified and, like for BAF155, the majority of these are in the MarR-like domain (Fig. 3b). The argument that the N-terminal module is functionally important and these mutations are tumour-promoting is reinforced by the results of a study modelling cancer-driver events that identified a variant in the MarR-like domain of BAF155^20^.

Overall, for BAF155, 14% of the mutants in the databases are predicted to be deleterious/cancer-driver mutants, while for BAF170 it is 38.5%. It is interesting to note that there are almost three times more predicted driver mutants in BAF170 that is expressed upon differentiation than in BAF155, which is found in complexes that are known to drive proliferation.

### A potential site on BAF155 for small-molecule drugs targeting

**Fig. 4:**
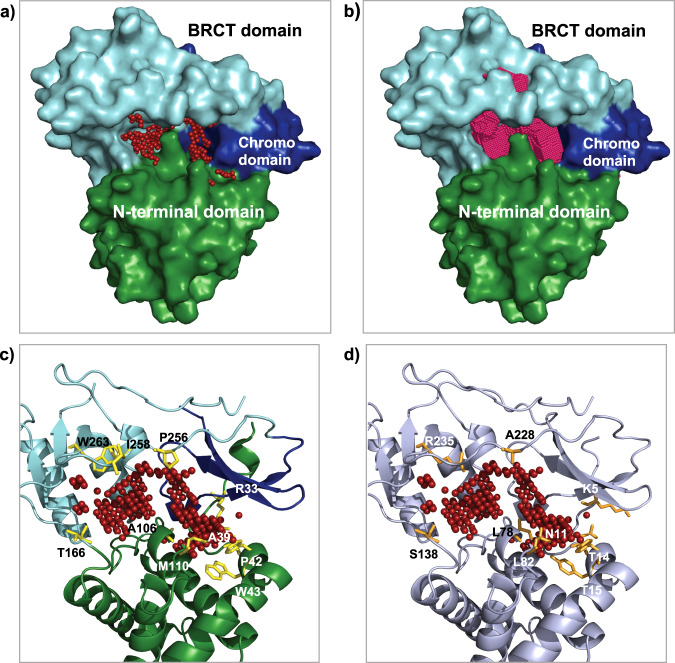
Computationally identified small-molecule-binding pockets as potential druggable sites. **a** Surface representation of the X-ray crystal structure of the N-terminal module of BF155 with the red dots indicating the binding site identified by SiteMap. **b** Binding site identified by DoGSite Scorer highlighted in pink. **c** Cartoon representation of the structure with the red dots indicating the binding site identified by SiteMap: the residues of the pocket on BAF155 that differs from BAF170 are labelled and highlighted in yellow (stick representation). **d** Highlighted in orange are the labelled residues (stick representation) on the carton representation of the homology model of BAF170 that differs from BAF155.

Attempts to target BAF155 have been stymied to date by a lack of small-molecule-binding sites. The programs Schrodinger SiteMap^21^ and DoGSiteScorer^22^ both identify the cleft between the domains as an amenable pocket for small-molecule binding (Fig. 4a, b): SiteMap DScore of 0.98 and DoGSiteScorer Drug Score of 0.81.

Sites at domain–domain interfaces amenable for small-molecule binding have been identified in other epigenetic regulators^23^, and it has been suggested that they could be exploited when so-called “epigenetic readers domains” are not themselves suitable for ligand binding. The site has a reasonable size and very good enclosure comparable to drug-binding sites in kinases, and an acceptable hydrophilic and hydrophobic properties compared to PPI-binding sites targeted to date. The sequence variation within this pocket between BAF155 and BAF170 suggests that it may be possible to create paralogue-selective molecules (Fig. 4c, d). Molecules targeting this site can be used as chemical probes to determine its function and as components of PROTACs to target BAF155/BAF170 mutant cancers.

## Methods

### Cloning expression and purification

The DNA encoding residues 28–303 of BAF155 was amplified from human cDNA by PCR and cloned into a vector based on pRSETA (Invitrogen) that expresses proteins fused to the lipoyl domain of *Bacillus stearothermophilus* dihydrolipoamide acetyltransferase with an N-terminal His6-tag. The native BAF155 module was expressed in *Escherichia coli* C41(DE3) in 2xTY media and induced by the addition of IPTG (1 mM final concentration). The cells were harvested by centrifugation, disrupted by sonication and the cell debris were removed by centrifugation at 20,000 rpm for 30 min. The protein was purified by Ni-NTA affinity chromatography and then dialysed overnight in the presence of TEV protease, which cleaves the BAF155 module from the lipoyl domain. A second affinity chromatography step was carried out to remove the lipoyl domain and the BAF155 module was further purified by gel filtration on a Superdex 75 HR column. K-MOPS minimal media^24^ was used for seleno-methionine labelling of the module. Cells were grown to an OD600 of 0.6 after which 100 mg/l of DL-Seleno-methionine (Sigma), 100 mg/l lysine, threonine and phenylalanine, leucine, isoleucine and valine were added as solids. IPTG (1 mM final concentration) was then added after a further 20 min and cells were grown for a further 16 h at 20 °C. Isotopically labelled proteins for NMR spectroscopy were prepared by growing cells in K-MOPS minimal media containing ^15^NH_4_Cl and/or [^13^C]-glucose. Seleno-methionine (SeMet) and isotopically labelled proteins were purified as described above.

### Crystallization

The protein was concentrated to 16 mg/ml and dialysed into 10 mM Tris, pH 7.0, 100 mM NaCl for crystallization. Native and SeMet BAF155 was crystallized at 16 mg/ml by sitting-drop vapour diffusion. Several conditions yielded crystals, both for the native and SeMet-labelled proteins, with 0.2 M magnesium formate (pH 5.9), 20% PEG 3350 yielding the best diffracting crystals subsequently used for data collection. The crystals were flash-frozen in liquid nitrogen after the addition of glycerol to 20% while leaving the other components of the mother liquor at the same concentration.

### Structure solution and refinement

SeMet BAF155 crystals belonging to space group P2_1_ were used to obtain phase information using the I03 beamline at Diamond Light Source, Oxford (UK). Data were obtained from 1800 images collected at 0.9755 Å with 0.1° increments at 100 K/−173.5 °C (wavelength of data collection 0.975456 Å). All images were integrated using XDS^25^ and scaled using SCALA^26^. Phases were obtained using Phaser SAD^27^ in the CCP4i software^28^ in combination with PARROT^29^ and SHELXD^30^. The initial output was subsequently built using BUCCANEER^31^ and further refined using iterative rounds of COOT^32^ and PHENIX^33^. Two molecules of BAF155 were observed in the asymmetric unit of the P2_1_ SeMet SAD dataset.

Percentage of residues in the ‘most favoured region’ of the Ramachandran plot: 97.93, 0.00; and percentage of outliers (Molprobity Clash Score): 7.22.

A native dataset, containing 1800 images collected at 0.1° increments (wavelength of data collection 0.939273 Å), was collected at beamline I03 of the Diamond Light Source at 100 K/−173.5 °C. The structure of native BAF155 module was obtained using molecular replacement from a refined structure of the protein obtained from the SeMet SAD data. All structures were refined using iterative rounds of COOT^32^ and PHENIX^33^.

Percentage of residues in the ‘most favoured region’ of the Ramachandran plot: 97.94, 0.00; and percentage of outliers (Molprobity Clash Score): 5.05.

Cell constants and crystallographic data, and details of the refined models shown in Table 1.

### NMR binding studies

NMR measurements were made using a Bruker DRX600 spectrometer equipped with a triple-resonance cryoprobe at 25 °C. NMR samples were typically 0.5 mM in 90% H_2_O, 10% D_2_O, containing 20 mM potassium phosphate, pH 6.5, 100 mM NaCl and 5 mM β-mercaptoethanol. Backbone assignments were carried using HNCO, HN(CA)CO, HNCA, HNCACB, CBCA(CO)HN 3D heteronuclear NMR experiments on ^2^H-, ^13^C- and ^15^N-labelled samples using standard Bruker pulse programs. Topspin (Bruker) was used for data processing and SPARKY was used for data analysis. The ability of the module to binding to mono, di and tri methyl-lysine was assessed by recording ^1^H-^15^N HQSC spectra of ^15^N-labelled protein with and without the addition of a five-fold excess of the amino acid.

### Conservation analysis

A multiple alignment of sequences of BAF155 and BAF170 homologues from different model organisms was prepared using Clustal Omega^34^. The ConSurf^17^ server was used to calculate conservation scores, using default values and the Bayesian method.

### Mutational analysis

Missense mutations within the N-terminal modules of BAF155 and BAF170 were extracted from the cBioPortal^35^ and the COSMIC^36^ databases. Only mutations from tumour samples were considered. To assess the effects of mutations in the equivalent module in BAF170 (which has 66% sequence identity to that in BAF155) we used the BAF155 structure to generate a homology model using the One-to-One Threading option in PHYRE2^37^ (the resulting model had a 100% confidence score). The program Missense3D^19^ was used to evaluate the effect of mutations on the stability of the modules. Variants were judged to have an effect on protein function if they are non-conservative substitutions of surface residues with ConSurf scores of 8 or 9.

### Reporting summary

Further information on research design is available in the Nature Research Reporting Summary linked to this article.

## Supplementary information

Supplementary Information

Reporting Summary

## Data Availability

The coordinate of the crystal structures are deposited in the Protein Data Bank (PBD), and the accession identifiers are 6YXO and 6YXP. The NMR assignments are deposited in the Biological Magnetic Resonance Bank BMRB, and the accession identifier is 50830. All the other data are available from the corresponding author on reasonable request.
