# Supplementary material for: SWI/SNF subunit BAF155 N-terminus structure informs the impact of cancer-associated mutations and reveals a potential drug binding site
Source: Commun Biol. 2021 May 5;4:528. doi: 10.1038/s42003-021-02050-z (PMC8099880; doi:10.1038/s42003-021-02050-z)
Supplement: Supplementary file 1 — Supplementary Information [file 42003_2021_2050_MOESM1_ESM.pdf]

The SWI/SNF subunit BAF155 N-terminus structure informs the impact of cancer-associated mutations and reveals a potential drug binding site.

Mark D. Allen[1], Stefan M.V. Freund[1], Mark Bycroft[1§] and Giovanna Zinzalla[2*]

1. UKRI MRC Laboratory of Molecular Biology, Cambridge, United Kingdom.

2. Microbiology, Tumor and Cell Biology (MTC) Department, Karolinska Institutet, Stockholm, Sweden

*corresponding author: Giovanna Zinzalla, giovanna.zinzalla@ki.se

§ current address: Department of Pharmacology, University of Cambridge, Cambridge, United Kingdom.

This file includes:

Supplementary Figures 1- 4

Supplementary Tables 1 and 2

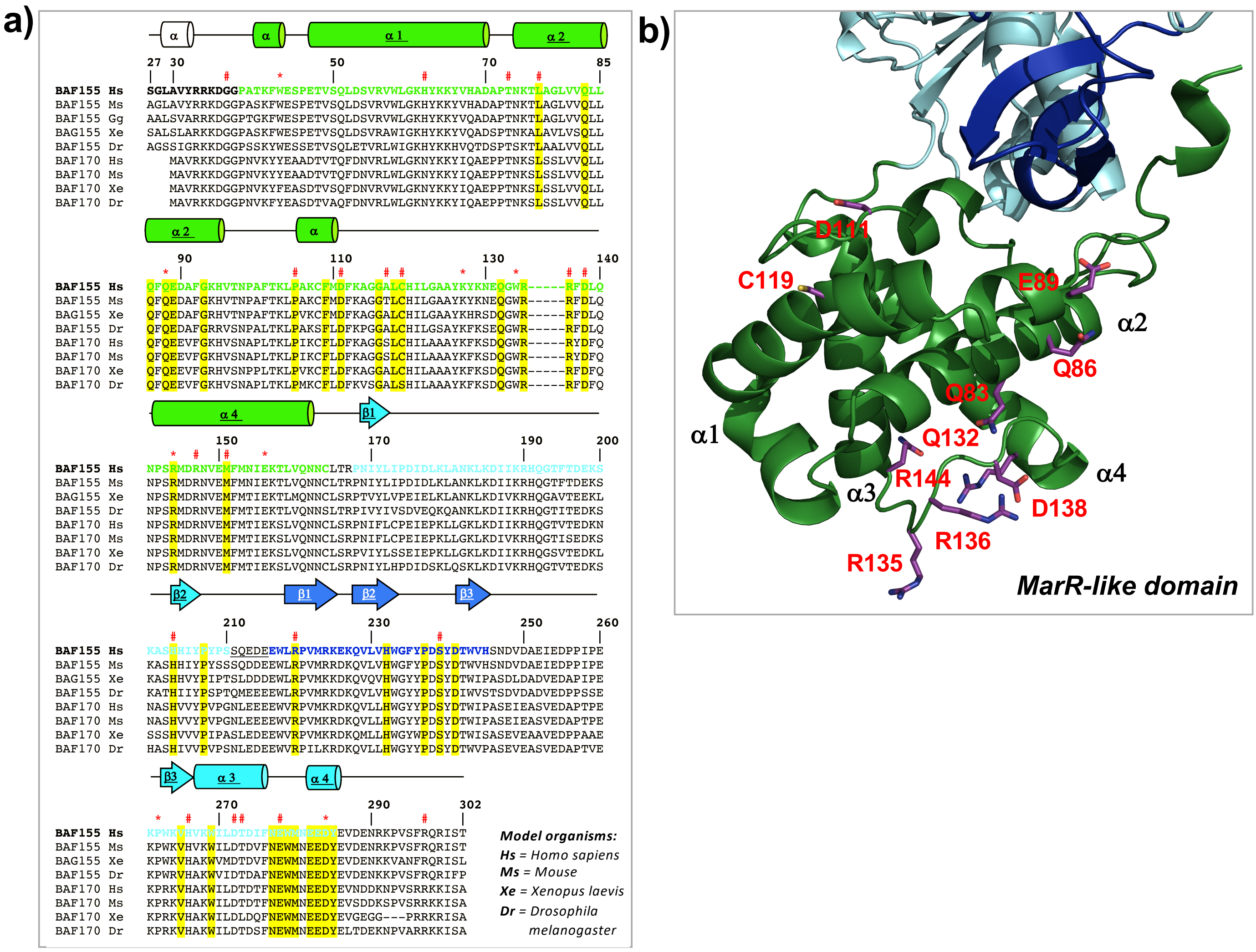

Supplementary Fig. 1. Sequence conservation

a) Sequence alignments of BAF155 and BAF170 with schematic representation of secondary structure. Highly-conserved residues (ConSurf score = 9) are highlighted in yellow; structural damaging mutations of BAF155 are highlighted with a red star, and for BAF170 with a red hash key. **b)** Highly-conserved surface residues (sidechains in purple in stick representation) in the MarR-like domain (in green, cartoon epresentation).

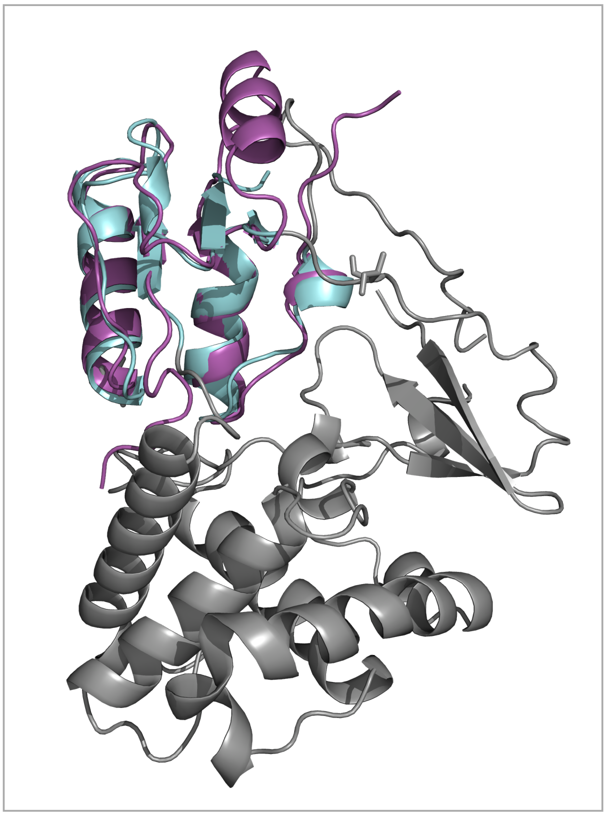

Supplementary Fig. 2. **Structural similarities to the BRCT domain.**
Overlay of the cartoon representations of the BRCT domain structure (in cyan) of BAF155 and of XRCC1 (in purple, PDB ID = 1CDZ).

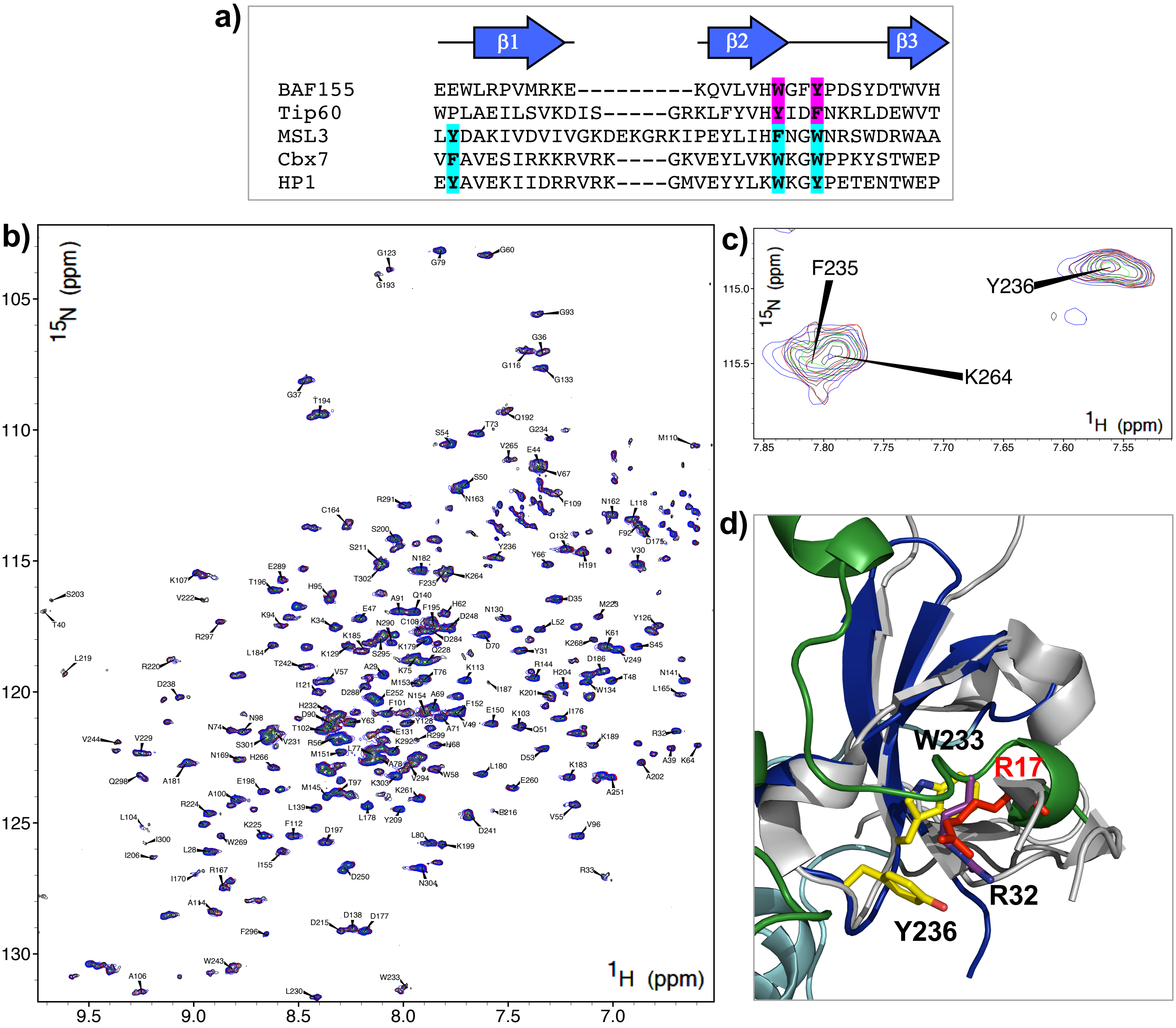

Supplementary Fig. 3. NMR binding studies to mono-, di and tri-methylated lysines to the chromodomain.

a) Sequence alignments of human chromodomains of the same family of BAF155 (Tip60 and MSL3) and from other families (Cbx7 and HP1). Highlighted in cyan the three residues that form the methyl-lysine “aromatic cage”, in magenta the only two present in BAF155 and Tip60. **b)** Overlay of annotated ^{1}H,^{15}N HSQC spectra of BAF155 N-terminal module with mono- (red), di- (blue), tri- (green) methylated lysines and without (black). **c)** Expanded region of the spectra showing residue Y236 part of the “aromatic cage”. **d)** Overlay of the carton representation of the chromodomains in BAF155 (blue) and TIPS60 (grey), with labelled and highlighted in yellow in stick representation the residues of the ”aromatic cage” on BAF155, and the arginine residues blocking the binding of methyl lysine in each of the domains (R17 highlighted in red on Tip60, R32 highlighted in purple on BAF155, both in stick representation).

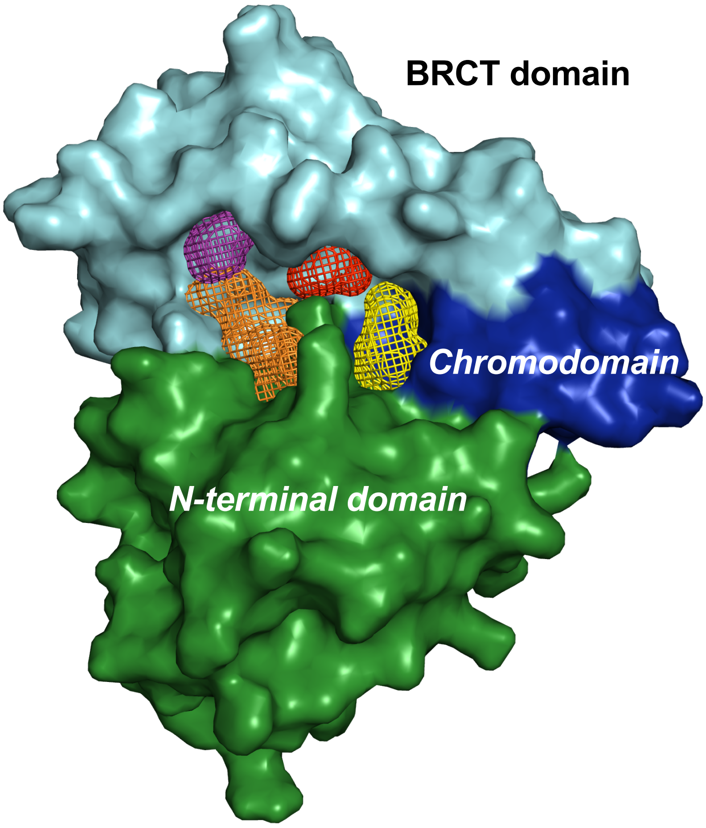

Supplementary Fig. 4. Computationally identified peptide binding pockets. Surface representation of the N-terminal module with in green the MarR-like domain, blue, the chromodomain and in cyan the BRCT domain with mesh representation the four main sites identified by PeptiMap for peptide binding with scoring from 1 to 4 (orange = site 1, yellow = site 2, red = site 3 and purple site 4).

Supplementary Table 1. BAF155 mutations in cancer from the cBioPortal and the COSMIC databases.

Entry	BAF155 mutant[a]	Cancer type	Missense3D structural change prediction[f]	ConSurf score
1	P38L	Lung SCC[b]	Neutral	8
2	P38L	Cutaneous Melanoma	Neutral	8
3	A39V	cSCC[c]	Neutral	8
4	K41T	Uterine Endometrioid Carcinoma	Neutral	6
**5**	**W43C**	**Cutaneous Melanoma**	**Damaging**	**4**
6	E44D	cSCC[c]	Neutral	8
7	P46Q	Cutaneous Melanoma	Neutral	1
8	A69V	Nasopharyngeal Carcinoma	Neutral	5
9	V82L	Lung carcinoma	Neutral	3
10	L84F	Cutaneous Melanoma	Neutral	7
**11**	**Q88L**	**Lung Adenocarcinoma**	**Damaging**	**9**
12	H95N	Liver cancer	Neutral	4
13	V96I	Gastric cancer	Neutral	5
14	A100T	Colorectal cancer	Neutral	4
15	F109L	Stomach Adenocarcinoma	Neutral	9
16	G115E	Acral Melanoma	Neutral	6
17	G115E	Cutaneous Melanoma	Neutral	6
18	I121L	Hepatocellular Adenoma	Neutral	8
19	L122F	Uterine Carcinosarcoma	Neutral	7
**20**	**K129T**	**Breast Cancer**	**Neutral**	**8**
**21-22**	**W134L**	**Twice, Thyroid Carcinoma**	**Neutral**	**8**
23	Q140H	Colon Adenocarcinoma	Neutral	7
24	P142Q	Cutaneous Melanoma	Neutral	6
25	P142T	Cutaneous Melanoma	Neutral	6
**26**	**R144Q**	**Rectal Adenocarcinoma**	**Neutral**	**9**
27	M145I	Cutaneous Melanoma	Neutral	6
28	R147G	Renal Clear Cell Carcinoma	Neutral	7
29	R147H	Stomach Adenocarcinoma	Neutral	7
**30**	**E156K**	**Skin Cancer, Non-Melanoma**	**Damaging**	**8**
31	D175G	Uterine Endometrioid Carcinoma	Neutral	1
32	L180S	Cutaneous Melanoma	Neutral	2
33	L184F	Lung Adenocarcinoma	Neutral	5
34	R190Q	UCEC[d]	Neutral	5
35	F195C	Gastric Adenocarcinoma	Neutral	2
36	T196M	Urothelial Carcinoma	Neutral	4
37	E198D	Glioblastoma Multiforme	Neutral	4
38	E198Q	Lung SCC[b]		4
39	P208Q	Lung Adenocarcinoma	Neutral	4
40	P208L	Breast Invasive Lobular Carcinoma	Neutral	4
41	E226Q	Upper Tract Urothelial Carcinoma	Neutral	6
42	L230V	LGGs[e]	Neutral	5
43-44	D250G	Twice, Breast Invasive Ductal Carc.	Neutral	5
45	A251V	SCC[b]	Neutral	1
46	E254K	Urothelial carcinoma	Neutral	6
47	I258V	Lung Adenocarcinoma	Neutral	1
48	I258V	UCEC[d]	Neutral	1
49	K261N	UCEC[d]	Neutral	1
50	K261Q	Colon Adenocarcinoma	Neutral	1
**51**	**P262Q**	**Uterine Serous Carcinoma**	**Damaging**	**1**
52	F276L	Lung SCC[b]	Neutral	6
53	D284E	UCEC[d]	Neutral	9
**54**	**D284N**	**Urothelial carcinoma**	**Damaging**	**9**
**55**	**D284N**	**Metastatic breast cancer**	**Damaging**	**9**
56	D288N	Ewing Sarcoma	Neutral	5
57	R297H	Gastric cancer	Neutral	6
58	Q298R	Breast Invasive Ductal Carcinoma	Neutral	2
59-60	R299Q	Twice, Breast Invasive Ductal Carc.	Neutral	7
61	R299Q	Breast Mixed Ductal and Lobular Carc.	Neutral	7
62	R299Q	UCEC[d]	Neutral	7
63	K303N	UCEC[d]	Neutral	5

Notes: (a) We have extracted all the missense mutations only from tumour samples in the structure region for BAF155; and then potential driver mutants (in bold and underlined) have been selected based on two criteria: 1. Structurally damaging based on Missense3D; 2. A non-conservative mutation at a residue that scores 8 or 9 in ConSurf. (b) Squamous Cell Carcinoma. (c) Cutaneous Squamous Cell Carcinoma. (d) Uterine Corpus Endometrial Carcinoma. (e) Low Grade Gliomas (brain tumours). (f) A homology model of the structure of BAF170 was generated using the One-to-One Threading software of Phyre2 server.

Supplementary Table 2. BAF170 mutations in cancer from the cBioPortal and the COSMIC databases.

Entry	BAF170 mutant[a]	Cancer type	Missense3D structural change prediction[e]	ConSurf score
1-2	V3A	Twice, Breast Invasive Ductal Carc.	Neutral	1
**3**	**G9S**	**Glioblastoma Multiforme**	**Damaging**	**5**
4	T22N	Liver Cancer	Neutral	2
5	A18V	Head and Neck Squamous Cell Carc.	Neutral	1
6	A18V	Breast Invasive Ductal Carcinoma	Neutral	1
7	D19N	UCEC[b]	Neutral	6
8	Q23K	Prostate Adenocarcinoma	Neutral	6
9	R28W	Malignant melanoma	Neutral	7
**10**	**N34S**	**Intestinal Type Stomach Adenocarc.**	**Neutral**	**8**
11	E42K	Urothelial carcinoma	Neutral	7
**12**	**T45I**	**cSCC[c]**	**Neutral**	**8**
**13**	**L49P**	**Gastric cancer**	**Damaging**	**8**
14	Q55H	UCEC[b]	Neutral	8
15	L56V	UCEC[b]	Neutral	6
16	P72L	Lung SCC[d]	Neutral	5
17	P72L	cSCC[c]	Neutral	5
**18**	**P77L**	**Biliary Tract Cancer**	**Damaging**	**8**
**19**	**P77L**	**Prostate Adenocarcinoma**	**Damaging**	**8**
**20**	**D83H**	**Lung Adenocarcinoma**	**Neutral**	**9**
21	K85E	Breast Invasive Ductal Carcinoma	Neutral	7
**22**	**S89F**	**UCEC[b]**	**Damaging**	**3**
**23**	**S89P**	**Stomach Adenocarcinoma**	**Damaging**	**3**
**24**	**C91F**	**Stomach Adenocarcinoma**	**Damaging**	**8**
25	K101R	Colon cancer	Neutral	8
26	D103N	Gastric cancer	Neutral	6
**27**	**R108C**	**UCEC[b]**	**Neutral**	**9**
**28**	**R108L**	**Breast Invasive Ductal Carc.**	**Neutral**	**9**
**29**	**D110N**	**Colorectal Adenocarcinoma**	**Neutral**	**9**
**30**	**D110N**	**UCEC[b]**	**Neutral**	**9**
**31**	**R119C**	**Cutaneous Melanoma**	**Neutral**	**8**
**32**	**M123R**	**Bladder Urothelial Carcinoma**	**Damaging**	**8**
33	T126I	Cutaneous Melanoma	Neutral	1
34	S130F	Cutaneous Melanoma	Neutral	3
35	R139Q	Colorectal Adenocarcinoma	Neutral	1
36	G154V/R	Lung Adenocarcinoma	Neutral	1
37	K161N	Glioblastoma Multiforme	Neutral	4
38	N172K	Papillary Renal Cell Carcinoma	Neutral	1
39	N173D	Breast Invasive Ductal Carcinoma	Neutral	1
40	D170E	Pulmonary Carcinoid	Neutral	3
**41-42**	**H176N**	**Twice, Cutaneous Melanoma**	**Damaging**	**9**
43	P182S	cSCC[c]	Neutral	1
44	G183W	Papillary Renal Cell Carcinoma	Neutral	1
45	V191L	Liver cancer	Neutral	6
**46**	**R192Q**	**Colon Adenocarcinoma**	**Damaging**	**9**
47	K196N	Breast Invasive Ductal Carcinoma	Neutral	3
**48**	**S211I**	**UCEC[b]**	**Neutral**	**9**
49	W215R	Prostate Adenocarcinoma	Neutral	5
50	A218V	Gastric Cancer	Neutral	1
51	E222Q	Breast Invasive Ductal Carcinoma	Neutral	4
52	P231A	Breast Invasive Ductal Carcinoma	Neutral	1
53	P231S	Glioblastoma	Neutral	1
**54**	**H238Y**	**Cutaneous Melanoma**	**Damaging**	**6**
55	D244N	Rectal Adenocarcinoma	Neutral	8
**56**	**D244G**	**UCEC[b]**	**Neutral**	**8**
**57-58**	**T245I**	**Twice, Cutaneous Melanoma**	**Damaging**	**6**
59	T247I	Desmoplastic Melanoma	Neutral	2
**60**	**E250K**	**Breast Invasive Lobular Carcinoma**	**Damaging**	**9**
61	R268C	Breast Invasive Ductal Carcinoma	Neutral	1
62	R268C	Breast Mixed Ductal and Lobular Carc.	Neutral	1
63	R268C	Colorectal Adenocarcinoma	Neutral	1
64	R268C	UCEC[b]	Neutral	1
**65**	**R269L**	**Lung SCC[d]**	**Damaging**	**8**

Notes: (a) We have extracted all the missense mutations only from tumour samples in the structure region for BAF155; and then potential driver mutants (in bold and underlined) have been selected based on two criteria: 1. Structurally damaging based on Missense3D; 2. A non-conservative mutation at a residue that scores 8 or 9 in ConSurf.(b) Squamous Cell Carcinoma. (c) Cutaneous Squamous Cell Carcinoma. (d) Uterine Corpus Endometrial Carcinoma (e) Low Grade Gliomas (brain tumours). (f) A homology model of the structure of BAF170 was generated using the One-to-One Threading software of Phyre2 server.
