# Supplementary material for: SWI/SNF subunit BAF155 N-terminus structure informs the impact of cancer-associated mutations and reveals a potential drug binding site
Source: Commun Biol. 2021 May 5;4:528. doi: 10.1038/s42003-021-02050-z (PMC8099880; doi:10.1038/s42003-021-02050-z)
Supplement: Supplementary file 2 — Reporting Summary [file 42003_2021_2050_MOESM2_ESM.pdf]

nature research

Corresponding author(s): Giovanna Zinzalla
Last updated by author(s): Mar 16, 2021

Reporting Summary

Nature Research wishes to improve the reproducibility of the work that we publish. This form provides structure for consistency and transparency in reporting. For further information on Nature Research policies, see our Editorial Policies and the Editorial Policy Checklist.

Statistics

For all statistical analyses, confirm that the following items are present in the figure legend, table legend, main text, or Methods section.

n/a	Confirmed	
☒	☐	The exact sample size (n) for each experimental group/condition, given as a discrete number and unit of measurement
☒	☐	A statement on whether measurements were taken from distinct samples or whether the same sample was measured repeatedly
☒	☐	The statistical test(s) used AND whether they are one- or two-sided<br>*Only common tests should be described solely by name; describe more complex techniques in the Methods section.*
☒	☐	A description of all covariates tested
☒	☐	A description of any assumptions or corrections, such as tests of normality and adjustment for multiple comparisons
☒	☐	A full description of the statistical parameters including central tendency (e.g. means) or other basic estimates (e.g. regression coefficient) AND variation (e.g. standard deviation) or associated estimates of uncertainty (e.g. confidence intervals)
☒	☐	For null hypothesis testing, the test statistic (e.g. F, t, r) with confidence intervals, effect sizes, degrees of freedom and P value noted<br>*Give P values as exact values whenever suitable.*
☒	☐	For Bayesian analysis, information on the choice of priors and Markov chain Monte Carlo settings
☒	☐	For hierarchical and complex designs, identification of the appropriate level for tests and full reporting of outcomes
☒	☐	Estimates of effect sizes (e.g. Cohen's d, Pearson's r), indicating how they were calculated

Our web collection on statistics for biologists contains articles on many of the points above.

Software and code

Policy information about availability of computer code

Data collection	X-ray Crystallography: I03 beamline at Diamond Light Source, Oxford (UK). NMR: Bruker DRX600 spectrometer equipped with a triple-resonance cryoprobe, and Bruker TopSpin Software for data collection and analysis.
Data analysis	NMR: Bruker TopSpin Software. X-ray Crystallography: XDS22, SCALA23. Phaser SAD24 in the CCP4i software, PARROT26 and SHELXD27. BUCCANEER, COOT and PHENIX. Conservation analysis: Clustal Omega and ConSurf server was used to calculate conservation scores, using default values and the Bayesian method from the server. Mutational analysis: cBioPortal1515 and the COSMIC16 databases; only mutations from tumor samples were considered. Structural analysis of mutation effect: Missense3D. Computational software to assess potential peptide binding: PeptiMap (analysis conducted using the software default settings). Computational softwares to assess potential small-molecule ligand binding: Schrodinger SiteMap and DoGSiteScorer ((analysis conducted using the software default settings with both). ALL the softwares, databases etc. have been described in published literature and referenced accordingly in the manuscript.

For manuscripts utilizing custom algorithms or software that are central to the research but not yet described in published literature, software must be made available to editors and reviewers. We strongly encourage code deposition in a community repository (e.g. GitHub). See the Nature Research guidelines for submitting code & software for further information.

Data

Policy information about availability of data

All manuscripts must include a data availability statement. This statement should provide the following information, where applicable:

- Accession codes, unique identifiers, or web links for publicly available datasets
- A list of figures that have associated raw data
- A description of any restrictions on data availability

The coordinate of the crystal structures are deposited in the PBD, and the accession identifiers are: 6YXO and 6YXP. The NMR assignments have been deposited in

the BMRB, and the accession identifier is: 50830.

Field-specific reporting

Please select the one below that is the best fit for your research. If you are not sure, read the appropriate sections before making your selection.

- [x] Life sciences
- [ ] Behavioural & social sciences
- [ ] Ecological, evolutionary & environmental sciences

For a reference copy of the document with all sections, see nature.com/documents/nr-reporting-summary-flat.pdf

Life sciences study design

All studies must disclose on these points even when the disclosure is negative.

Sample size	Not applicable.
Data exclusions	Not applicable.
Replication	Not applicable.
Randomization	Not applicable.
Blinding	Not applicable.

Reporting for specific materials, systems and methods

We require information from authors about some types of materials, experimental systems and methods used in many studies. Here, indicate whether each material, system or method listed is relevant to your study. If you are not sure if a list item applies to your research, read the appropriate section before selecting a response.

Materials & experimental systems

n/a	Involved in the study	
x		Antibodies
x		Eukaryotic cell lines
x		Palaeontology and archaeology
x		Animals and other organisms
x		Human research participants
x		Clinical data
x		Dual use research of concern

Methods

n/a	Involved in the study	
x		ChIP-seq
x		Flow cytometry
x		MRI-based neuroimaging
